# Draft Genome Sequences of Three Pseudomonas chengduensis Strains Isolated from Desert Soil in Morocco

**DOI:** 10.1128/mra.01082-22

**Published:** 2023-01-12

**Authors:** Houda Zouagui, M. W. Chemao-Elfihri, Amina Manni, Meryem Laamarti, Souad Kartti, Tarek Alouane, Lamiaa Lahlou, Oussama Benhrif, Loubna Temsamani, Jamal-Eddine Eljamali, Abdelkarim Filali-Maltouf, Azeddine Ibrahimi, Laila Sbabou

**Affiliations:** a Laboratory of Microbiology and Molecular Biology, Faculty of Sciences, Mohammed V University, Rabat, Morocco; b Biotechnology Lab (MedBiotech), Bioinova Research Center, Rabat Medical & Pharmacy School, Mohammed V University, Rabat, Morocco; c Mohammed VI University of Health Sciences, UM6SS, Casablanca, Morocco; Wellesley College

## Abstract

We report the draft genome sequences of three Pseudomonas chengduensis strains isolated from the sand dunes of the Merzouga (MDMC17 strain) and Erg Lihoudi (MDMC216 and MDMC224 strains) regions in the Moroccan desert. These bacteria are able to tolerate the harsh environmental conditions of the desert ecosystem.

## ANNOUNCEMENT

Deserts make up 33% of the total land area, and life in these regions is constantly challenged by diverse environmental extremes such as water scarcity, salinity, temperature fluctuations, and high UV radiation levels (1). Like all life forms, the desert microbial community has developed special adaptation strategies that are important for survival in this harsh habitat. The genus Pseudomonas is composed of Gram-negative, aerobic, rod-shaped, mobile bacteria (2). Pseudomonas bacteria are widely distributed in nature and have been isolated from various environments (3). Currently, the genus comprises 572 species with validly published names in the List of Prokaryotic Names with Standing in Nomenclature (LPSN) (4). In this study, we report the draft genome sequences of three Pseudomonas chengduensis strains, MDMC17, MDMC216, and MDMC224, isolated from the sand dunes of Merzouga and Erg Lihoudi in the Moroccan Sahara. To our knowledge, this is the first report of the genome sequence of Pseudomonas chengduensis strains isolated from a desert ecosystem.

The sand samples were collected from 0- to 5-cm depth in the Merzouga (MDMC17) and Erg Lihoudi (MDMC216 and MDMC224) regions at the coordinates N31°6′33.6″, W3°58′42.675″, and N29°54′7.596″, W5°41′12.138″, respectively.

One gram of sand sample was suspended in 9 mL of physiological water. The suspension was agitated vigorously for 30 min and serially diluted (10^−3^ to 10^−7^). One hundred microliters from each dilution was plated in nutrient agar medium supplemented with 50 μg/mL of amphotericin B and incubated at 28°C for 2 days. The strains were purified and stored in liquid nutrient broth with 40% glycerol solution at −80°C.

Genomic DNA was extracted from a liquid nutrient broth following the phenol-chloroform method (5) and quantified using a Quantus fluorometer. A paired-end sequencing library was prepared with the Nextera XT DNA sample preparation kit (Illumina, Inc., San Diego, CA, USA) and sequenced on an Illumina MiSeq instrument using the MiSeq reagent kit v3 (600-cycle) (Illumina).

The quality control of the reads was assessed using FastQC v0.11.6 (6). Trimming and filtering of low-quality sequences were performed by Trimmomatic v0.39 (7). *De novo* assembly was performed by SPAdes v3.09.0 (8) for MDMC224 and by AbySS v2.02 (9) for MDMC17 and MDMC216. The total sequence lengths are 5,839,369 bp (MDMC17), 5,557,664 bp (MDMC216), and 5,591,703 bp (MDMC224). Further genomic data are provided in Table 1.

**TABLE 1:** Genomic features of the three strains

Feature^*a*^	MDMC17	MDMC216	MDMC224
No. of reads	1,769,626	2,511,788	1,751,142
No. of contigs	97	122	166
Genome coverage (×)	72.50	108	75
Contig *N*_50_ (bp)	122,034	99,417	242,468
Total sequence length (bp)	5,839,369	5,557,664	5,591,703
GC content (%)	62.56	62.61	62.54
No. of CDSs	5,319	5,058	5,138
No. of rRNAs	14	6	11
No. of tRNAs	65	46	60
No. of ncRNAs	5	5	5
No. of pseudogenes	97	87	101
ANIm best match (%)	97.03	97.03	97.02
dDDH best match (%)	73.50	73.30	73.30

a CDSs, coding DNA sequences; ncRNAs, noncoding RNAs.

**FIG 1:**
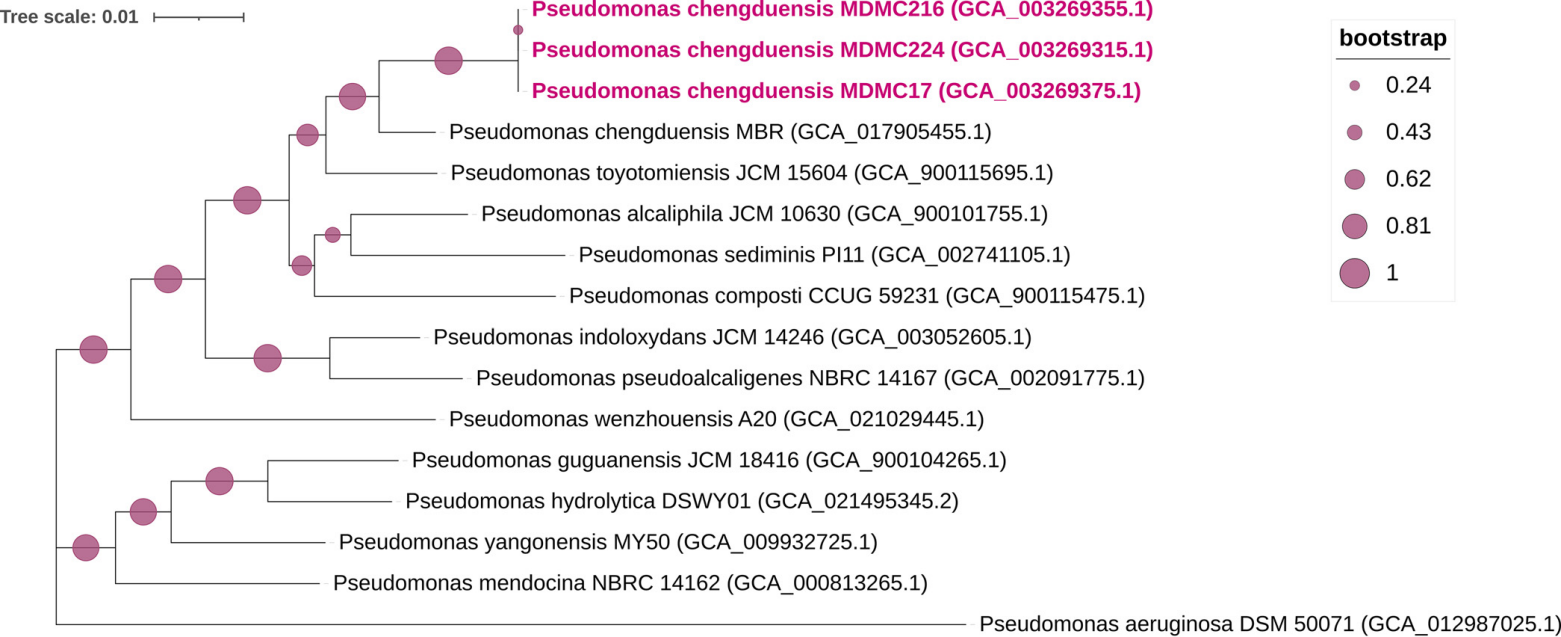
Multilocus sequence type phylogenetic tree inferred from maximum likelihood analysis of concatenated 16S rRNA, *gyrB*, *rpoB*, and *rpoD* sequences with 1,000 bootstrap iterations. Type strains of the closest Pseudomonas spp. were collected from the NCBI RefSeq database, and Pseudomonas aeruginosa DSM 50071 (GCA_012987025.1) was used as outgroup.

Annotation was carried out using RAST server version 2.0 (10) and the NCBI Prokaryotic Genome Annotation Pipeline 5.3 (11). Strain identification was processed by the Type (Strain) Genome Server (TYGS) (12). Multilocus sequence analysis (MLSA) of four housekeeping genes (16S rRNA, *gyrB*, *rpoB*, and *rpoD*) was performed, and a maximum likelihood tree was constructed using MEGA X (13). The phylogenetic tree revealed that Pseudomonas chengduensis MDMC17, MDMC216, and MDMC224 were most closely related to Pseudomonas chengduensis MBR (JAAAMF000000000) (Fig. 1). The identification was confirmed by The average nucleotide identity based on MUMmer (ANIm) calculations using Pyani v0.2.9 (14), and digital DNA-DNA hybridization (dDDH) calculations using Formula 2 Genome-to-Genome Distance Calculator GGDC v2.1 (https://ggdc.dsmz.de/ggdc.php) (15). Pseudomonas chengduensis type strain MBR (JAAAMF000000000) showed the best-match average nucleotide identity (ANI) and dDDH values against the three isolates (Table 1). Default parameters were used for all of the previous software tools.

### Data availability.

The draft genome assemblies are available under the DDBJ/ENA/GenBank accession numbers QLYW00000000 (MDMC17), QLYV00000000 (MDMC216), and QLYU00000000 (MDMC224), with the BioSample accession numbers SAMN09380012 (MDMC17), SAMN09380013 (MDMC216), and SAMN09380014 (MDMC224). The raw reads were deposited in the NCBI Sequence Read Archive (SRA) under the BioProject accession number PRJNA886995, with the SRA accession numbers SRX17793472, SRX17793475, and SRX17793476 for MDMC17, MDMC216, and MDMC224, respectively.
